# Transcriptomic changes in the posterior pallium of male zebra finches associated with social niche conformance

**DOI:** 10.1186/s12864-024-10573-y

**Published:** 2024-07-15

**Authors:** Sepand Riyahi, Navina D. Liebermann-Lilie, Arne Jacobs, Peter Korsten, Uwe Mayer, Tim Schmoll

**Affiliations:** 1 Evolutionary Biology, Bielefeld University, Konsequenz 45, Bielefeld, 33615 Germany; 2 Department of Evolutionary Anthropology, University of Vienna, Djerassiplatz 1, Vienna, 1030 Austria; 3 Department of Animal Behaviour, Bielefeld University, Konsequenz 45, Bielefeld, 33615 Germany; 4 Institute of Biodiversity, Animal Health and Comparative Medicine, University of Glasgow, Glasgow, UK; 5 Department of Life Sciences, Aberystwyth University, Aberystwyth, UK; 6 Center for Mind/Brain Science, University of Trento, Piazza Manifattura 1, Rovereto, TN 38068 Italy; 7 Joint Institute for Individualisation in a Changing Environment (JICE), University of Münster and Bielefeld University, Bielefeld, Germany

**Keywords:** Aggression, Courtship, Male-male competition, Open Science Framework, Pre-registration, RNA-Seq, Social monogamy, Sperm competition, Transcriptomics, Weighted correlation network analysis

## Abstract

Animals plastically adjust their physiological and behavioural phenotypes to conform to their social environment—social niche conformance. The degree of sexual competition is a critical part of the social environment to which animals adjust their phenotypes, but the underlying genetic mechanisms are poorly understood. We conducted a study to investigate how differences in sperm competition risk affect the gene expression profiles of the testes and two brain areas (posterior pallium and optic tectum) in breeding male zebra finches (*Taeniopygia castanotis*). In this pre-registered study, we investigated a large sample of 59 individual transcriptomes. We compared two experimental groups: males held in single breeding pairs (low sexual competition) *versus* those held in two pairs (elevated sexual competition) per breeding cage. Using weighted gene co-expression network analysis (WGCNA), we observed significant effects of the social treatment in all three tissues. However, only the treatment effects found in the pallium were confirmed by an additional randomisation test for statistical robustness. Likewise, the differential gene expression analysis revealed treatment effects only in the posterior pallium (ten genes) and optic tectum (six genes). No treatment effects were found in the testis at the single gene level. Thus, our experiments do not provide strong evidence for transcriptomic adjustment specific to manipulated sperm competition risk. However, we did observe transcriptomic adjustments to the manipulated social environment in the posterior pallium. These effects were polygenic rather than based on few individual genes with strong effects. Our findings are discussed in relation to an accompanying paper using the same animals, which reports behavioural results consistent with the results presented here.

**Supplementary Information:**

The online version contains supplementary material available at 10.1186/s12864-024-10573-y.

## Introduction

To maximise fitness in heterogeneous environments, animals must adjust their phenotypes to ever-changing environmental conditions [1–3]. This includes matching their physiological and behavioural phenotypes to conform with the current social environment. Such *social niche conformance* [4] is likely associated with phenotypic variation among individuals, especially in response to social conflicts [4, 5]. The degree of sexual competition is a critical part of the social environment to which animals may adjust their phenotypes [6–8]. The degree of sexual competition is shaped by the density of potential mating partners and same‐sex competitors and leads to the sexual selection of competitive traits [9–12]. In mating systems that are not strictly genetically monogamous, sexual selection also operates through sperm competition [13, 14] and/or cryptic mate choice [15, 16].

Males represent the more competitive sex in most animal mating systems [17, 18]. Male investment in competitive traits is costly [19], promoting the evolution of individual phenotypic plasticity [3] of male competitive traits in response to variation in the social environment. For example, the level of (perceived) sperm competition affects ejaculate size [20] and composition [21] and has been hypothesised to affect costly behavioural traits such as aggression [22, 23]. Although there is substantial evidence suggesting that males are sensitive to key socio-sexual factors such as rival density, the underlying molecular mechanisms that enable these phenotypically plastic responses remain largely unclear.

Changes in morphological, physiological or behavioural phenotypes in response to the social environment are mediated by mechanisms that alter how genes are expressed without altering the underlying genetic sequence [24, 25]. Gene expression analysis based on RNA sequencing provides a powerful tool for directly investigating these phenomena [26]. For example, differential gene expression studies were successfully used in studies on female mating preferences [27], elevated nest site competition [28], sex-differential ornament expression [29], the re-modelling of male mating tactics [30], male territory defence [31] and individual variation in male sexual competitive ability [32]. However, despite some illustrative examples (see e.g [33–35].), it remains generally unclear how differential gene expression allows for individual phenotypic plasticity in male competitive traits.

**Fig. 1:**
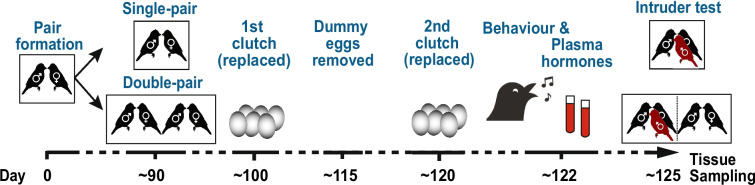
Schematic representation of the experimental procedures (modified from [41]). One day after the first egg of the 2nd clutch was laid, we recorded the birds’ behaviours in the breeding cage (2 h)*.* We quantified male courtship behaviour as total singing duration (min) consisting of song directed to the social mate (or the non-focal female in the case of the Double-pair treatment group) and undirected song (for details, see Supplementary Table S5 in [41]). On the following day, we took blood samples, obtaining plasma T and CORT levels. Four days after the replacement clutch was initiated, we staged a standardised intruder test, in which we measured the focal males’ aggressive responses against an unfamiliar stimulus male introduced into their breeding cage (10 min). Aggression was defined as the total time (min) the focal and the stimulus male spent in any aggressive interactions (for details, see Supplementary Table S6 in [41])

In this pre-registered study [36], we report on socially sensitive gene expression in male zebra finches (*Taeniopygia castanotis,* formerly called *T. guttata*), a monogamous passerine bird with biparental care. Zebra finches show extra-pair mating in captivity [37] and have frequently been used as a model in studies on mate choice [37, 38] and sperm competition [39, 40]. We investigated whether phenotypic plasticity due to variation in sperm competition risk and associated social stimulation is reflected in their gene expression. We conducted RNA-sequencing comparing the gene expression profiles of male zebra finches across two experimental groups exposed to different social environments: Single-pair males (low sperm competition risk) *versus* Double-pair males (elevated sperm competition risk, see Fig. 1). The effects of this treatment on the males’ behavioural and hormonal profiles have been reported in a complementary paper [41] (see Table 1 for a summary of the results from this paper).

**Table 1:** Summary of predictions and results of behavioural and hormonal responses to experimentally manipulated sperm competition risk analysed in [41]

Trait	Prediction	Result	Response variable	Breeding stages	Data provenance
Singing	Single-pair < Double-pair	Single-pair > Double-pair	Total time spent singing (min)	Before & during egg-laying	Breeding cage observations
Allopreening	Single-pair < Double-pair	Single-pair = Double-pair	Total time spent allopreening (min)	Before & during egg-laying	Breeding cage observations
Copulations (within-pair)	Single-pair < Double-pair	Single-pair = Double-pair	Within-pair copulation attempts (no.)	Before & during egg-laying	Breeding cage observations
Proximity to social mate	Single-pair < Double-pair	Single-pair > Double-pair	Proportion of time in close proximity (%)	Before & during egg-laying	Breeding cage observations
Chasing	Single-pair < Double-pair	Single-pair > Double-pair	Total duration of chasing (min)	During egg-laying	Intruder test
Aggression total	Single-pair < Double-pair	Single-pair > Double-pair	Total duration of agonistic interactions (min)	During egg-laying	Intruder test
Plasma testosterone (T)	Single-pair < Double-pair	Single-pair = Double-pair	T concentration	Before & during treatment	Blood plasma
Plasma corticosterone (CORT*)	Single-pair ≠ Double-pair	Single-pair = Double-pair	CORT concentration	Before & during treatment	Blood plasma

^*^CORT was a pre-registered dependent variable, but without a directional hypothesis

As reported in our accompanying paper [41], zebra finch males adjusted their behavioural phenotypes to the manipulated social environment, indicating social niche conformance [4]. However, contrary to our pre-registered expectations [36], there was no evidence that these behavioural adjustments were related to the elevated sperm competition risk (Table 1). We expected the Double-pair males to show more courtship displays, including songs directed towards their social mate, more affiliative and copulation behaviour with their social mate and more aggression towards competitors compared to Single-pair males [36]. On the contrary, males of the Double-pair treatment group decreased courtship rates (song), and when confronted with an unfamiliar intruder, they responded less aggressively than Single-pair males [41]. No other behavioural measures revealed significant treatment effects (see [41] for all details).

These unexpected effects suggest that in our study, we observed adjustment of behaviour to social factors other than sperm competition risk, like mild social isolation. Single-pair males were deprived of direct interactions with other males and females, which are typical of this highly social colonial breeder [42]. Consequently, Single-pair males reacted with increased aggression to the appearance of a new male. On the contrary, males in the Double-pair treatment showed less within-pair interactions (i.e., time spent courtship singing and time spent near their social mates) and aggression towards an unfamiliar intruder [41]. Double-pair males were used to the presence of another male and thus more tolerant towards novel male intruders. This is in line with studies showing that zebra finches with reduced exposure to social partners react more aggressively towards competitors than those held in larger groups [43, 44] (for similar results in mammals and fish, see, e.g [45, 46].). Please note that the experimental setting was designed to study the subtle effects of increased sperm competition risk by a mild manipulation of the group constellation in the close proximity of the focal males. All animals were in acoustical and visual contact also with other conspecifics in other breeding cages in the same room. This was done to mimic more natural conditions for sperm competition risk of this colonial bird. While the subtle social manipulation was not enough to induce robust changes in the testis physiology, it was enough to induce social niche conformance. To conclude, although not in line with our preregistered expectations, the social treatment induced behavioural phenotypic plasticity.

The current paper focuses on the gene expression profiles in different tissues of the same zebra finch males. For each male, we obtained the transcriptome from one of the testes and two brain regions (posterior pallium and optic tectum). We decided to study gene expression of the testes because of their central role in traits linked to sperm competition (plasticity in testosterone profiles and ejaculate traits [47]). Although our behavioural results did not reveal clear signatures of sperm competition [41], we still hypothesised that potentially subtle effects of sperm competition could be reflected in testes transcriptomes. As for the brain, our pre-registered experimental design involved transcriptomic analysis of the septum [36], an area involved in regulating aggression [48–50]. For technical reasons, we could not extract a sufficient amount of RNA from this brain area. Thus, we analysed the transcriptome of the posterior pallium and the optic tectum.

We selected the posterior pallium, because it contains large portions of amygdala-homologue brain regions (arcopallium, posterior amygdala and nucleus taenia of the amygdala) [51, 52]. These regions are part of the social decision-making network [53], which modulates multiple social responses, including fear/aggression towards conspecifics. The optic tectum—the first station of the primary visual pathway of birds—we selected as a control region, which we expected to be unaffected by the social treatment. We thus studied the key tissues (testis and brain) implicated in regulating male competitiveness to shed light on the molecular mechanisms underlying social niche conformance. The large number of individual transcriptomes allowed us to conduct a differential expression analysis of individual genes between the two treatment groups without pooling the data of multiple individuals. Additionally, this large sample allowed us to perform a gene co-expression network analysis to study if groups of genes with highly correlated expression (co-expression modules) were affected by the experimental treatment [54].

## Results

### Distinct gene expression between the testis, posterior pallium and optic tectum

**Fig. 2:**
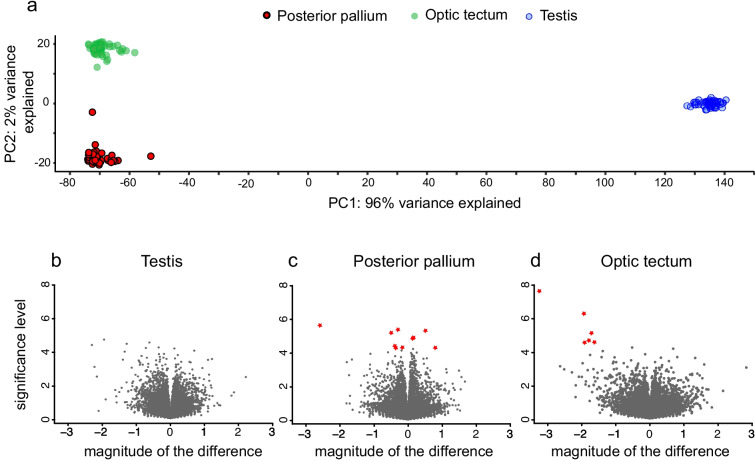
**a** Principal component plot showing differential gene expression across the three male zebra finch target tissues. Each dot represents a single sample from testis (blue), the posterior pallium (red with a black contour) and the optic tectum (green without a contour). **b-d** Volcano plots showing differential gene expression between the two treatment levels within each target tissue. On the x-axis is the magnitude (log twofold change) of the difference in gene expression between the two groups (higher values indicate higher expression in the Double-pair relative to the Single-pair level). The y-axis displays the significance (-log_10_ of the false discovery rate-corrected *p*-value) of the differential gene expression. Each dot represents a single gene. Significantly differentially expressed genes are indicated as red stars

We collected 180 tissue samples from the testis, posterior pallium and optic tectum of 60 male zebra finches (*n* = 30 males per treatment level). Not all samples provided sufficient RNA, though, resulting in overall 173 tissues samples from 60 individuals (testis Single-pair *n* = 29, Double-pair *n* = 30; posterior pallium Single-pair: *n* = 28, Double-pair: *n* = 29; optic tectum Single-pair: *n* = 28, Double-pair *n* = 29). Across the 173 tissue samples, a total of 21,040 genes annotated in the zebra finch genome [55, 56] were expressed. As expected, the overall gene expression patterns summarised by a principal component analysis clearly distinguished the three tissues (Fig. 2), revealing highly tissue-specific expression patterns. We thus performed gene (co-)expression analyses separately for each tissue. Specifically, we compared Single-pair and Double-pair males to identify genes and gene networks whose expression levels were sensitive to our experimental treatment.

### Differential gene expression in the testis, posterior pallium and optic tectum of Single- *versus* Double-pair males

At the single gene expression level, no treatment effects were found in the testis, and only a few treatment-sensitive genes were identified in the posterior pallium and optic tectum. Overall, 20,380 expressed genes were identified in the testis (TES). While the transcriptomic profiles differed substantially among individual males, analyses restricted to individual genes did not reveal any differential expression between Single- *versus* Double-pair males in the testis. In the posterior pallium (PAL), 10 of 18,917 identified genes were differentially expressed between Single-pair and Double-pair males (Table 2). Four genes had higher expression in Double- compared to Single-pair males, while six genes showed lower expression levels in the Double-pair treatment (Table 2). In the optic tectum (OT), six out of 19,030 genes had lower expression in Double-pair males than in Single-pair males (Table 2). One gene (predicted: *MTTP*) showed lower expression in both tectum and posterior pallium in Double-pair compared to Single-pair males (Table 2).

**Table 2:** Differentially expressed genes between Single- versus Double-pair males in the posterior pallium and optic tectum. The magnitude of the difference in expression is represented by log twofold change (log_2_FC). Higher values indicate higher expression in the Double-pair relative to the Single-pair treatment level. False discovery rate (FDR) refers to p-values that were adjusted for multiple comparisons. The significance level was set to FDR < 0.1

Gene symbol	Gene name	log_2_FC	FDR
**Pallium: higher expression in the Double-pair treatment level**			
MC5R	Melanocortin 5 receptor	0.519	0.031
GNA11	G Protein Subunit Alpha 11	0.163	0.044
RND3	Rho Family GTPase 3	0.138	0.044
Predicted: UGT8 (LOC100228968)	2-hydroxyacylsphingosine 1-beta galactosyltransferase	0.813	0.099
**Pallium: higher expression in the Single-pair treatment level**			
Predicted: MTTP (LOC105759397)	Microsomal triglyceride transfer protein large subunit	-2.596	0.031
Predicted: SMAD6 (LOC105758838)	Mothers against decapentaplegic homolog 6	-0.297	0.031
ZNRD2	Zinc Ribbon Domain Containing 2	-0.496	0.031
VAMP8	Vesicle Associated Membrane Protein 8	-0.388	0.099
TVP23A	Trans-Golgi Network Vesicle Protein 23 Homolog A	-0.165	0.099
MXD1	MAX Dimerization Protein 1	-0.353	0.099
**Tectum: higher expression in the Single-pair treatment level**			
Predicted: MTTP (LOC105759397)	Microsomal triglyceride transfer protein large subunit	-3.244	< 0.001
Uncharacterised (LOC115492340)	-	-1.937	0.005
PNMT	Phenylethanolamine N-methyltransferase	-1.715	0.055
Predicted: OASL1 (LOC100224927)	2'-5'-oligoadenylate synthase-like protein 1	-1.792	0.099
Predicted: IFIT5 (LOC100229421)	Interferon-induced protein with tetratricopeptide repeats 5	-1.628	0.099
Uncharacterised (LOC115492673)	-	-1.917	0.099

### Treatment-dependent expression of gene networks in the testis, posterior pallium and optic tectum

Complex remodelling of integrated phenotypes may be caused by concerted and potentially subtle expression changes of networks of co-expressed genes rather than by dramatic expression changes in single genes. The genes forming such co-expression networks are likely involved in the same or similar functional pathways [57, 58]. We thus applied weighted co-expression gene network analysis (WGCNA) to identify groups of genes (co-expression modules) that changed expression in response to the experimental social treatment [54].

**Fig. 3:**
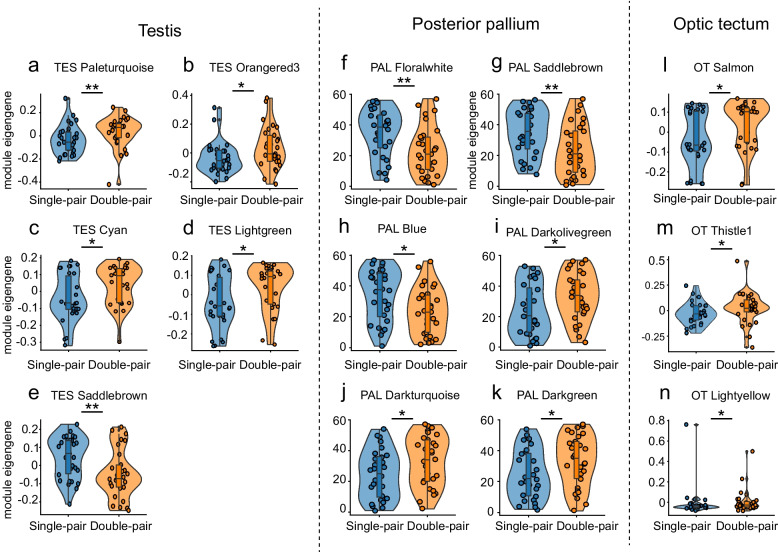
Gene co-expression network modules with significant differences between the Single-pair *versus* Double-pair treatment levels for the three target tissues. Each module represents a cluster of genes whose expression is highly correlated among individual transcriptomes (within tissues). Each dot represents the data from one individual male for that module (Single- and Double-pair levels are represented in blue and orange, respectively, and modules are labelled by the colour assigned by WGCNA software). Module eigengene, on the y-axis, is defined as the first principal component of the expression matrix of the corresponding module. The values indicate the extension of the module eigengene. Note that the y-axis scale differs across the panels (*p < 0.05; **p < 0.01). **a**-**e** Testis (TES), (**f**-**k**) Posterior pallium (PAL) and (l-n) Optic Tectum (OT)

In the testis, we detected 66 modules representing clusters of genes with highly correlated expression (Supplementary Data Table S1a; note that modules are named by arbitrary colours assigned by the WGCNA software). Five of these modules were differently expressed between the two treatment groups (Fig. 3a-e). These treatment-sensitive modules contained between 40 and 265 genes, of which 8–46 were classified as hub genes, a class of highly connected genes within a module (Table 3). The statistical results for all identified modules, including lists of identified genes and hub genes of each treatment-sensitive module, are summarised in Supplementary Table S1a-c.

**Table 3:** Gene co-expression modules with significant differences between Single- and Double-pair males in the testis (TES), posterior pallium (PAL) and optic tectum (OT)

Modules	Number of genes	Number of hub genes		Statistics
TES Paleturquoise	128		18	F_(1,57)_ = 7.180, *p* = 0.010
TES Orangered3	40		8	F_(1,57)_ = 5.663, *p* = 0.021
TES Cyan	265		38	F_(1,57)_ = 4.654, *p* = 0.035
TES Lightgreen	210		46	F_(1,57)_ = 4.581, *p* = 0.037
TES Saddlebrown	140		24	F_(1,57)_ = 7.472, *p* = 0.008
PAL Floralwhite	108		28	F_(1,55)_ = 8.327, *p* = 0.006
PAL Saddlebrown	173		59	F_(1,55)_ = 7.251, *p* = 0.009
PAL Blue	1986		179	F_(1,55)_ = 6.180, *p* = 0.016
PAL Darkolivegreen	160		160	F_(1,55)_ = 4.506, *p* = 0.038
PAL Darkturquoise	193		47	F_(1,55)_ = 4.137, *p* = 0.047
PAL Darkgreen	210		28	F_(1,55)_ = 4.066, *p* = 0.049
OT Salmon	255		40	F_(1,55)_ = 5.054, *p* = 0.029
OT Thistle1	48		10	F_(1,55)_ = 4.430, *p* = 0.040
OT Lightyellow	190		5	F_(1,55)_ = 4.066, *p* = 0.049

In the posterior pallium, we identified 49 gene co-expression modules (Supplementary Data Table S2a). Six modules showed significantly different expressions between the two treatment groups (Fig. 3f-k). These modules contained between 108 and 1986 genes, of which 28–179 were classified as hub genes (see Table 3). The results for all identified modules in the posterior pallium, including the list of all genes contained in the modules and hub genes of treatment-sensitive modules, are summarised in Supplementary Table S2a-c.

In the optic tectum, 40 modules were identified (Supplementary Data Table S3a). Three modules were differently expressed between the treatment groups (Fig. 3l-n). These modules contained between 48 and 255 genes, of which 5–40 were classified as hub genes (see Table 3 for a summary of the treatment-sensitive modules and Supplementary Table S3a-c for an overview of all results, including the list of genes and hub genes).

### Robustness of treatment effects at the level of gene co-expression networks

**Fig. 4:**
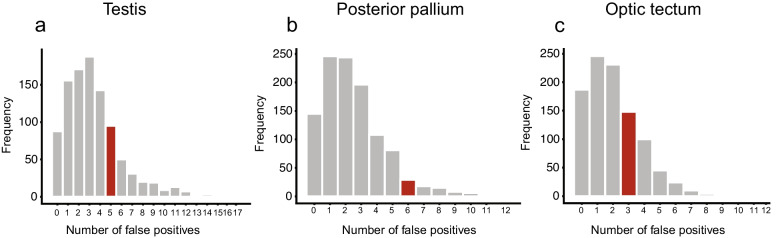
Results of a permutation test. The plots show the distribution of false positive results after 1000 trial shuffles. The frequency of false positives is on the y-axis, and the number of modules with false positive differences between the two treatment groups is on the x-axis. The red bar indicates the number of modules that showed significant treatment effects in our data. **a** Testis (**b**) Posterior pallium and (**c**) Optic Tectum

We conducted additional randomisation tests for each of the three tissues using a trial shuffle method to evaluate if the measured treatment effects could have emerged by chance. We shuffled trials for 1000 times. In each trial, the individual eigengene values of the gene co-expression modules were randomly assigned to the two treatment groups. ANOVA was performed to identify the number of gene co-expression modules with false positive treatment effects (Fig. 4). We aimed to identify how often it occurs by chance that 5 out of 66 modules in the testis, 6 out of 49 in the posterior pallium, and 3 out of 40 modules in the optical tectum are significantly different, as was the case in our real data.

Analysis of the testis revealed that in 95 out of 1000 permutations, five modules significantly differed between treatments (red bar in Fig. 4a). These are 9.5% of cases and suggest a false positive probability of p = 0.095. Thus, we conclude that the detected treatment effects in testis are likely false positives.

In the posterior pallium, six modules significantly differed between treatments in 29 out of 1000 permutations (2.9%, red bar Fig. 4b). Therefore, we consider our results in the pallium to be robust, with a probability of false positives of *p* = 0.029.

In the tectum, the analysis revealed that in 148 out of 1000 permutations (14.8%), 3 modules significantly differed between treatments (red bar in Fig. 4c). Thus, we conclude that the effects we observed in the tectum are likely false positives (*p* = 0.148).

### Functions of the treatment-sensitive gene co-expression modules in the pallium

To specify the biological processes associated with the genes of the six modules significantly affected by the experimental treatment in the pallium, we performed gene ontology (GO) enrichment analysis [59]. This analysis is based on functional annotations databases, reflecting the scientific community's current understanding of the functions associated with each gene [60].

Four out of the six significantly affected modules contained significantly enriched GO terms. All 55 GO terms identified for the Blue module in pallium (PAL Blue module) were significantly enriched. PAL Saddlebrown had 31 significantly enriched GO terms out of 4832 identified terms. PAL Darkturquoise contained five (out of 5,376), and PAL Darkgreen 6 (out of 3,870) significantly enriched GO terms. The other modules (PAL Floralwhite, containing overall 1,887 GO terms and PAL Darkolivegreen, containing 3,045 GO terms) did not present significantly enriched GO terms. The functional annotations of the top five significantly enriched GO terms for each module are summarised in Table 4 (for all other GO terms, see Supplementary Table S2d-i).

**Table 4:** Top five significant gene ontology (GO) terms for biological processes within the posterior pallium (PAL) modules that were significantly different between treatment levels. The number of significantly enriched GO terms are indicated in brackets for each module

Module	Top five terms	Term identity
PAL Floralwhite (0)	-	-
PAL Saddlebrown (31)	Oligodendrocyte differentiation Glial cell differentiation Gliogenesis Glial cell development Ensheathment of neurons	GO:0048709 GO:0010001 GO:0042063 GO:0021782 GO:0007272
PAL Blue (55)	Neuroactive ligand-receptor interaction Intrinsic component of membrane Integral component of membrane Signaling Cell communication	KEGG:04080 GO:0031224 GO:0016021 GO:0023052 GO:0007154
PAL Darkolivegreen (0)	-	-
PAL Darkturquoise (5)	Semaphorin receptor activity Transmembrane signaling receptor activity Molecular transducer activity Signaling receptor activity Neurotransmitter receptor activity involved in regulation of postsynaptic cytosolic calcium ion concentration	GO:0017154 GO:0004888 GO:0060089 GO:0038023 GO:0099583
PAL Darkgreen (6)	Meiosis I Meiosis I cell cycle process Reciprocal homologous recombination Homologous recombination Reciprocal meiotic recombination	GO:0007127 GO:0061982 GO:0140527 GO:0035825 GO:0007131

**Fig. 5:**
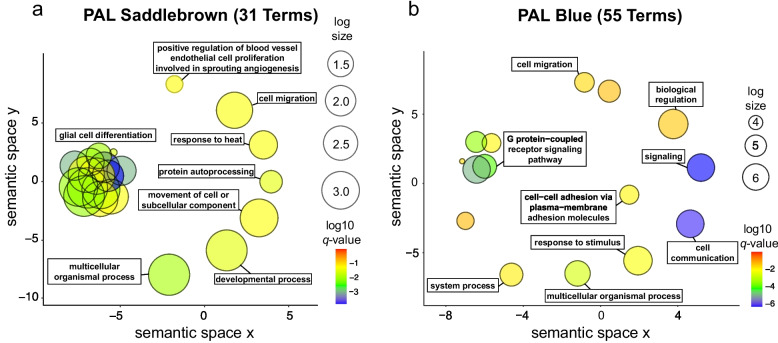
Semantic similarity scatterplots summarising lists of enriched Gene Ontology (GO) terms for two differentially expressed modules in the posterior pallium, which had a higher number of significantly enriched GO terms (number in brackets after the module name). Each bubble represents semantically closely related GO terms grouped by the semantic similarities of the genes’ functional annotations, thus summarising their underlying biology. The x- and y-axes represent a two-dimensional annotation space obtained by multi-dimensional scaling on a matrix of the semantic similarities of the GO terms (closer bubbles have higher semantic similarity). Clusters of bubbles are labelled for their most prominent function. Bubble sizes reflect the number of annotations for the GO terms (log size scale on the right). In contrast, their colour reflects the significance level (*q*-value colour heat scale on the bottom right, with blue indicating higher significance levels, based on log-transformed and FDR-corrected p-values). **a** Functions of the module PAL Saddlebrown (**b**) Functions of the module PAL Blue

The functions of the two modules with the highest numbers of significantly enriched GO terms for pallial tissue (PAL Saddlebrown and PAL Blue) have been further summarised as semantic space scatter plots (Fig. 5a-b). This revealed a macro cluster of functions related to the G protein coupled receptor signalling pathway in the PAL Blue module. In contrast, in the PAL Saddlebrown module, a predominance of functions related to glial cell differentiation was evident. Cell communication and signalling were significantly enriched in the PAL Blue but not in the PAL Saddlebrown module. Overall, most gene functions of the PAL Blue module seem to be related to neuronal processes and signalling. On the contrary, the PAL Saddlebrown module mainly identifies gene functions related to glial cells (see Table 4). The main functions shared by both modules are related to cell migration and multicellular organismal processing. As for the PAL Darkturquoise module genes, the main functions seem to be associated with the activity of different neural receptors. The PAL Darkgreen module’s functions mainly relate to meiosis (see Table 4).

## Discussion

In this pre-registered study [36], we hypothesised that elevated sperm competition risk experienced by Double-pair males would affect gene expression in the testis and brain compared to Single-pair males. Contrary to our expectations, we did not find transcriptomic adjustment clearly attributable to manipulated sperm competition risk. The differential gene expression analysis revealed treatment effects only in the posterior pallium (ten genes) and optic tectum (six genes). However, no treatment effects were detectable in the testis. Similarly, at the level of gene co-expression networks, we observed robust differences between the treatment groups only in the posterior pallium. On the other hand, effects suggested by our initial analysis in the testis and optic tectum might represent false positives, as revealed by randomisation tests. In the following, we discuss our results for each target tissue and in relation to the hormonal and behavioural results obtained from the same individuals, which were reported in detail in our complementary paper [41].

We expected our analysis to reveal differential gene expression between the two treatment groups in the testis, especially for candidate genes previously implicated in male-male competition [36]. The testes are responsible for sperm [61] but also androgen production, with downstream effects on tissues elsewhere, particularly the brain [62, 63]. The testes, thus, arguably constitute a target tissue of major importance for uncovering the effects of variation in sperm competition risk on gene expression. Contrary to our expectation, males who had the opportunity for extra-pair mating and faced higher sperm competition risk did not show clear changes in their testis transcriptomes. Our transcriptomic results here align with the behavioural and hormonal results [41] (see also Table 1), which show no evidence for treatment-induced changes in the male competitive traits studied. There are several possible explanations for why this was the case.

Although zebra finches have frequently been used to study sperm competition in the laboratory [64], they show strong monogamous pair bonds with low extrapair paternity levels. In the wild, the level of extrapair paternity has been investigated in two separate populations and was found to be less than 2% in both [65, 66]. Furthermore, the low level of sperm competition in the wild is consistent with the genetic architecture of the spermatozoa themselves [67] and the high variation in sperm morphology [68]. In line with this is the rather passive nature of competition in this species and the low levels of testosterone expressed by zebra finches [41, 68–70]. The testosterone levels increase slightly in male zebra finches during their partner's fertile window [47, 71]. However, this is mainly related to optimising the production of sperm and likely does not affect sperm competition or competition in general. Moreover, zebra finches are colonial breeders. They prefer to breed near other conspecifics [42, 72], and breeding is often socially facilitated [73]. Thus, the effects of sperm competition in this species can be considered rather minor. At the same time, we used a simple design to compare single pairs with double pairs. Although we manipulated the opportunity for extra-pair mating, the presence of only one other breeding pair may not have been a strong enough stimulus to induce robust changes in the male competitive traits we were studying.

Nevertheless, our treatment affected some behavioural traits and the transcriptomes in both brain tissues studied, indicating responses in neural functions that regulate behavioural phenotypes. This provides valuable evidence on the brain gene expression patterns underlying behavioural adjustment to different social environments. Both brain tissues contained some differently expressed individual genes. Although their number was rather low (ten in the posterior pallium and six in the optic tectum), this provided a first indication of social treatment-dependent transcriptomic changes in the brain. However, differences were much more substantial at the level of gene co-expression networks. Six (out of 49) co-expression modules in the posterior pallium, containing 2,830 genes overall, showed differences between treatments. The randomisation test revealed that the effects found in the pallium are robust. These results imply that male phenotypic adjustment to the social environment was based on polygenic processes rather than strong effects of a few individual genes. The enriched functional annotations of two gene co-expression modules, PAL Blue and PAL Darkturquoise, indicate modifications related to neural functions in the posterior pallium. The enriched functions of the module PAL Saddlebrown showed changes related to the development and differentiation of glial and endothelial cells. Together this indicates that the social treatment induced modifications of neural processes, which were accompanied by changes in the glial cells. Changes in endothelial cells may also indicate cerebrovascular plasticity, which is needed to adjust blood supply to changes in the metabolic demands of neural and glial cells [74].

Such social experience-dependent modulation of gene expression in the posterior pallium was expected as many of the functions of this large brain region regulate social behaviours at different levels. Specifically, the samples from the posterior pallium contained a large portion of the posterior nidopallium, almost the entire arcopallium, the posterior amygdala and the nucleus taenia of the amygdala. At large, the arcopallium and nucleus taenia of the amygdala in birds regulate fear [75]. More specifically, this region responds to novel stimuli, such as exposures to novel environments [76] or novel objects [77] and first encounters with conspecifics in naïve birds [78]. Moreover, the nucleus taenia of the amygdala is part of the social behaviour network, which is shared among all vertebrates. It comprises interconnected areas rich in sex steroid receptors and is implicated in a range of social behaviours, including aggression [53, 79, 80].

In our accompanying paper, we report that Single-pair males showed higher levels of aggression towards an unfamiliar intruder and spent more time in close proximity to their social mates compared to Double-pair males [41]. Hence, our social treatment led to changes in the neural mechanisms that control the response to familiar and unfamiliar conspecifics. The transcriptomic data of the present paper suggests that at least part of these neural changes occurred in the posterior pallium and are likely linked to the amygdaloid functions of this area. We also found that Single-pair males sang less compared to Double-pair males, which indicates a modification of the neural process of their song system. The posterior pallium contains the robust nucleus of the arcopallium (RA), which is the primary output of the telencephalic song system in songbirds [81]. The RA sends outputs to brain-stem regions that innervate the avian vocal organ (syrinx). During song production, RA neurons are active and are believed to encode the acoustic properties of song syllables [82–84]. It is thus likely that our transcriptomic data also reflect some adjustments of the song production system.

While the results we observed when analysing this large section of the brain are extremely helpful for our explorative purpose, it remains largely unclear which specific brain processes and regions were affected by our treatment. The arcopallium is a large brain region, which in zebra finches has been divided into six major domains with twenty distinct sub-regions [85]. The arcopallium receives inputs from numerous brain areas and is a major source of descending sensory and motor projections. It can thus be considered a key brain region of the avian forebrain [85]. Likewise, the avian posterior nidopallium is a large brain region supporting many functions, from working memory [86], executive functions [87] and visual categorisation [88] to sexual imprinting in zebra finches [89]. Which of these brain functions were affected by our treatment needs further investigation.

We also found subtle changes in gene expression in the optic tectum, which was unexpected. The optic tectum, located in the dorsal midbrain of birds, is the primary recipient of around 80% of retinal inputs. Its main function is the generation of orienting responses to stimuli of interest, especially when they are moving [90]. These responses are considered innate or reflexive [91]. At this early stage of visual processing, we did not expect to find plastic adjustments to our social treatment. Contrary to our expectation, however, six genes were differently expressed between the treatment levels. While the number of affected genes is very low and needs further confirmation, the potential effects indicate that some lasting adjustment to the social environment could be present in the optic tectum. This is an interesting finding, which may be explained by the organisation of the tectofugal visual pathway. In birds, the tectofugal visual pathway stretches from the retina to the optic tectum, then to the nucleus rotundus in the thalamus, before reaching the entopallium in the forebrain. The entopallium sends projections to higher telencephalic regions, including the arcopallium [92]. The arcopallium, in turn, projects back on the optic tectum, completing a tecto-tectal loop [93]. It is thus possible that the plastic changes found in the posterior pallium induced changes in the optic tectum through this loop. The tectofugal visual pathway in birds is involved, among other things, in perception and attention to object and shape information [88, 94, 95]. The projections from arcopallium to tectum could thus mediate preferential attention to social stimuli, such as the visual appearance of male and female conspecifics. Another potentially important source of social adjustment in this area could be related to acoustical communication. The tectal tissue we extracted contained the nucleus mesencephalicus lateralis (MLd, MLv), a part of the auditory pathway in zebra finches [96]. This could indicate alterations in basic auditory attention mechanisms and social acoustic stimuli detection. The neural basis of this interesting phenomenon needs to be further investigated.

## Conclusions

Our study indicates the importance of the social environment as a driver of phenotypic plasticity in zebra finch males, not only on the behavioural [41], but also at the gene-expression level. Manipulating the opportunity for extra-pair mating and, thereby, the risk of sperm competition, failed to induce changes in gene expression in the testes. However, the social treatment affected the gene expression in the brains of male zebra finches. The changes in the posterior pallium can be associated with behavioural adjustments of male zebra finches to changes in the social environment. These social treatment effects were foremost apparent at the level of gene co-expression networks in the posterior pallium, indicating that biological traits subject to phenotypic adjustment to the social environment are based on polygenic processes rather than a few individual genes with large effects. While many questions remain, the present study opens new doors for expanding our understanding of the mechanisms behind social niche conformance.

## Methods

### Experimental approach and general methods

This study has been pre-registered with the Open Science Framework [36], and its general methods, behavioural phenotyping and hormone profiling have been described in detail in a complementary paper [41]. Here we only summarise the main aspects in reference to [41]. In brief, zebra finch breeding pairs were randomly allocated to one of two experimental treatment groups, Single-pair *versus* Double-pair (Fig. 1). Thus, we exposed the breeding pairs to two different social environments reflecting two different levels of sperm competition risk and social stimulation. Only the males of the Double-pairs were exposed to sperm competition risk and direct physical interaction with another breeding-pair.

At the start of the experimental treatments (ca. 89 days after pair formation), we transferred Single-pair males to new breeding cages together with their established social mates. Double-pair males were transferred to a double-sized breeding cage, together with their social mate and an additional unrelated and unfamiliar social pair. All birds were fitted with three additional leg rings of the same colour to allow for individual identification during behavioural observations. The provision of nest boxes and nesting material stimulated the birds to start breeding. We stimulated breeding pairs to produce two consecutive clutches to give the focal males enough time to respond to the experimental treatment. For both clutches, plastic dummy eggs replaced the eggs on the day they were laid. We removed the first dummy clutch 15 days after the first egg of a clutch was laid, to induce the production of the second clutch (replacement clutch). Data and sample collection from the males were scheduled with regard to the timing of laying of the replacement clutch (see Fig. 1). This procedure ensured that the timing of data collection was standardised according to the reproductive cycle of each breeding pair, and that females were still receptive when male competitive behavioural traits were recorded. Multiple phenotypic traits were recorded per individual (see Table 1). Finally, on the fourth day after the first egg of the replacement clutch was laid, immediately after an intruder test (reported in [41]), we sacrificed 30 males from each treatment level (Double-pair and Single-pair) for gene expression analyses targeting testes and two brain areas (posterior pallium and optic tectum).

### Tissue dissection

Focal males were sacrificed by decapitation. The brain was extracted from the skull within 3–5 min, snap-frozen on dry ice and stored at -80 °C until further processing. This was followed by extraction of the left testis (which usually represents the bigger testis [97]). The right testis was not used but reserved for flow cytometric analysis of cell ploidy patterns. After the mass of the extracted testis was measured to a precision of 0.1 µg on a standard laboratory scale (Sartorius Quintex 124-1S), it was cut into two approximately equal pieces. One half was incubated for 24 h in RNA*later*™ Stabilization Solution (Invitrogen, catalogue number: AM7021) and stored at -80 °C until RNA extraction. The other half was reserved for flow cytometric analysis of cell ploidy patterns.

We extracted the posterior pallium and optic tectum, which deviates from what was declared in the pre-registration [36], as we initially planned to analyse the transcriptome of the septum (a core region of the social behaviour network). However, the small septum region did not provide sufficient RNA for individual-level transcriptome analysis. We thus extracted larger brain regions with similar functions and added a control region from the sub-pallium (the optic tectum). Before extraction, the brains were stored for two hours at -20 °C. This preserved brain anatomical structures while softening the frozen tissue to allow dissection. The brain regions of interest were then extracted from the right hemispheres on ice using binoculars. The left hemispheres were not used. The anatomical areas were located and delineated based on visual observation of anatomical landmarks as referenced in the zebra finch brain atlas [98]. The pallial samples were taken from a region approximating the one between anterior A 1.08 to posterior P 0.18 and including the entire tissue lateral to L 1.0. It thus included a large portion of the posterior nidopallium, almost the whole arcopallium, the posterior amygdala and the nucleus taenia of the amygdala. We did not include the most dorsal parts of the telencephalon to avoid inadvertently sampling from the hippocampal formation (HF) and the high vocal centre (HVc). The tectal samples were extracted approximating a region from A 2.43 to A 1.35 and including the entire tissue lateral to L 3.0. The samples thus included all the layers of the optic tectum as well as parts of the torus semicircularis (TOS), substantia grisea et fibrosa periventriculare (SGP), nucleus mesencephalicus lateralis (MLd, MLv) and nucleus isthmi (IPC, IM). The isolated brain regions were incubated in RNA*later*™ for 24 h at 4 °C and then stored at -80 °C until RNA extraction.

### RNA isolation and sequencing

Total RNA was extracted using the RNeasy Mini Kit (Qiagen, catalogue number 74106) for total RNA extractions. In addition to the protocol supplied with this kit, RNA samples were purified with RNase-Free DNase Set (Qiagen, catalogue number 79254) to eliminate possible DNA contamination. After RNA extraction, the quality of extracted RNA samples was tested with the 2100 Bioanalyzer System (Agilent). This revealed that for all of the samples the RNA integrity numbers (RIN) were > 8, indicating high-quality RNA. Extracted total RNA samples were subsequently sent to the Beijing Genomics Institute (BGI, www.genomics.cn) for complementary DNA library construction and mRNA sequencing using the DNBseq platform. mRNA-seq libraries were prepared using the BGI in-house library preparation kit (BGI, www.genomics.cn) and sequenced with 100 bp paired-end reads. Read filtering was performed by SOAPnuke [99]. Quality control of raw reads was performed with FastQC v0.11.9 [100]. On average 74 million clean reads were obtained for each sample, with an average Phred quality score of 37 after trimming.

### Genome mapping

We used hisat2 v.2.2.1 [56] for mapping the reads to the zebra finch reference genome (GenBank assembly accession*:* GCF_008822105.2). We used strict mapping options (–no-discordant –no-mixed) to avoid mismapping. On average 85% of the reads per sample were uniquely mapped to the genome. The gtf-file of the zebra finch reference genome, in combination with the mapped reads, was used to generate the reads count using htseq-count v.0.6.1 [55], a Python-based framework to analyse sequencing data (settings used: –format bam –order pos –mode union –stranded no –minaqual 1 –type exon).

### Differential gene expression

Differential expression analyses (DEGs) concerning the experimental treatment were conducted with the R package DEseq2 (v.1.26.0) [101] separately for each tissue (using the model: ~ Treatment). The false discovery rate (FDR) *q*-value for the DEseq2 was set as default (FDR < 0.1). Genes with fewer than ten total reads across all samples for any tissue were filtered out. For visualisation, the data were normalised using the standard formula (log_2_(counts + 1)), as suggested by the manual of the DEseq2 (v.1.26.0) [101] package. We used the clustering of samples by Principal Component Analysis to visualise gene expression in different target tissue types. Volcano plots were used to visualise differential gene expression between treatments separately for each tissue.

### Weighed correlation network analysis (WGCNA)

Weighted correlation network analysis (WGCNA) [54] was used to identify clusters (modules) of highly co-expressed genes. Modules are composed of genes with a similar co-expression pattern, creating a network centred on the so-called hub genes (﻿genes with a relatively high number of connections with other genes within the co-expression network are denoted as network hubs [58]). Since genes act together in shared pathways and networks, this analysis provides additional insights into even subtle but network-wide expression changes related to the experimental treatment [57, 58]. Genes with fewer than ten total reads across all samples for any tissue were filtered out here, too. We used the variance-stabilizing transformed data (generated with the VST function of the DEseq2 R package) as input for WGCNA.

The number of genes included in this analysis for the posterior pallium, optic tectum and testis were 18,917, 19,030 and 20,380, respectively. We created signed networks by selecting soft threshold power (β) using the pickSoftThreshold function (for posterior pallium β = 7, for tectum β = 6, for testis β = 7). We kept modules with a minimum module size of 30 genes. A threshold of 0.25 was used to merge modules. We identified hub genes in significantly associated modules as those with gene significance values above 0.2 and module memberships above 0.6. We tested for differences in module eigengene expression between treatments using ANOVA. All statistical analyses were performed using R [102].

### Functional annotation analysis

To specify the biological processes associated with the genes of the modules significantly affected by the experimental treatment we performed gene ontology (GO) [59] and KEGG (Kyoto Encyclopaedia of Genes and Genomes) [103] enrichment analysis, also called over-representation analysis (ORA). This was done using the WEB-based toolkit g:Profiler [104] (https://biit.cs.ut.ee/gprofiler/gost; g:Profiler version *e104_eg51_p15_3922dba* [October 2021]). The analysis is based on gene annotation terms, to determine differences in the processes/functions associated with a subset of genes (in this case, those of each co-expression module), compared to the functional annotations of the full list of all known zebra finch genes for the same tissue. To do so, we ran a functional enrichment analysis on the list of candidate genes (un-ordered list) for each significant module from the WGCNA, in relation to the experimental treatment, with an FDR < 0.05. The main results were further summarised by semantic similarity scatterplots visualised by REVIGO [105].

### Supplementary Information

Supplementary Material 1. Table S1: Supplementary information for genes and gene networks expressed in testis tissue. a) Detected gene networks, including ANOVA statistics for comparing Single-pair *versus* Double-pair males. Significantly differentially expressed modules are denoted in red. b) Full lists of genes of detected gene networks. Significantly differentially expressed modules are denoted in red. c) Hub genes of gene networks with significantly differential expression in relation to the experimental treatment. d) Gene ontology terms for the module TES Saddlebrown. e) Gene ontology terms for the module TES Paleturquoise. f) Gene ontology terms for the module TES Orangered3. g) Gene ontology terms for the module TES Cyan. h) Gene ontology terms for the module TES Lightgreen.

Supplementary Material 2: Table S2: Supplementary information for genes and gene networks expressed in posterium pallium tissue. a) Detected gene networks, including ANOVA statistics for comparing Single-pair*versus* Double-pair males. Significantly differentially expressed modules are denoted in red. b) Full lists of genes of detected gene networks. Significantly differentially expressed modules are denoted in red. c) Hub genes of gene networks with significantly differential expression in relation to the experimental treatment. d) Gene ontology terms for the module PAL Floralwhite. e) Gene ontology terms for the module PAL Saddlebrown. f) Gene ontology terms for the module PAL Blue. g) Gene ontology terms for the module PAL Darkolivegreen. h) Gene ontology terms for the module PAL Darkturquoise. i) Gene ontology terms for the module PAL Darkgreen.

Supplementary Material 3: Table S3: Supplementary information for genes and gene networks expressed in optic tectum tissue. a) Detected gene networks, including ANOVA statistics for comparing Single-pair *versus* Double-pair males. Significantly differentially expressed modules are denoted in red. b) Full lists of genes of detected gene networks. Significantly differentially expressed modules are denoted in red. c) Hub genes of gene networks with significantly differential expression in relation to the experimental treatment. d) Gene ontology terms for the module OT Salmon. e) Gene ontology terms for the module OT Thistle1. f) Gene ontology terms for the module OT Lightyellow.

## Data Availability

RNA sequencing data of this study is available through the European Nucleotide Archive (https://www.ebi.ac.uk/ena/browser/home). Study ID: PRJEB63186 (ERP148339).

## References

[1] Via S (1993). Adaptive phenotypic plasticity: target or by-product of selection in a variable environment?. Am Nat.

[2] Pigliucci M. Phenotypic Plasticity: Beyond Nature and Nurture. Baltimore: The John Hopkins University Press. 2001.

[3] Nussey DH, Wilson AJ, Brommer JE (2007). The evolutionary ecology of individual phenotypic plasticity in wild populations. J Evol Biol.

[4] Trappes R (2022). How Individualized Niches Arise: Defining Mechanisms of Niche Construction, Niche Choice, and Niche Conformance. Bioscience.

[5] Bergmüller R, Taborsky M (2010). Animal personality due to social niche specialisation. Trends Ecol Evol.

[6] Schradin C (2013). Intraspecific variation in social organization by genetic variation, developmental plasticity, social flexibility or entirely extrinsic factors. Philos Trans R Soc Lond B Biol Sci.

[7] Montiglio P-O, Wey TW, Sih A (2017). Effects of the group’s mix of sizes and personalities on the emergence of alternative mating systems in water striders. Behav Ecol.

[8] Fraser BA, Janowitz I, Thairu M, Travis J, Hughes KA (2014). Phenotypic and genomic plasticity of alternative male reproductive tactics in sailfin mollies. Proc Biol Sci.

[9] Darwin C, Bonner JT, May RM. The Descent of Man, and Selection in Relation to Sex. Princeton, New Jersey: Princeton University Press. 1981.

[10] Andersson M (1994). Sexual Selection.

[11] Andersson M, Simmons LW (2006). Sexual selection and mate choice. Trends Ecol Evol.

[12] Jones AG, Ratterman NL (2009). Mate choice and sexual selection: what have we learned since Darwin?. Proc Natl Acad Sci U S A.

[13] Parker GA (1970). Sperm Competition and Its Evolutionary Consequences in the Insects. Biol Rev.

[14] Pitnick SS, Hosken DJ, Birkhead TR. Sperm Biology: An Evolutionary Perspective. Amsterdam; London: Elsevier/Academic Press. 2009.

[15] Eberhard W (1996). Female Control: Sexual Selection by Cryptic Female Choice.

[16] Firman RC, Gasparini C, Manier MK, Pizzari T (2017). Postmating Female Control: 20 Years of Cryptic Female Choice. Trends Ecol Evol.

[17] Parker GA (2014). The sexual cascade and the rise of pre-ejaculatory (Darwinian) sexual selection, sex roles, and sexual conflict. Cold Spring Harb Perspect Biol.

[18] Janicke T, Häderer IK, Lajeunesse MJ, Anthes N (2016). Darwinian sex roles confirmed across the animal kingdom. Sci Adv.

[19] Dougherty LR (2021). Meta-analysis reveals that animal sexual signalling behaviour is honest and resource based. Nat Ecol Evol.

[20] Kelly CD, Jennions MD (2011). Sexual selection and sperm quantity: meta-analyses of strategic ejaculation. Biol Rev Camb Philos Soc.

[21] Hopkins BR (2019). Divergent allocation of sperm and the seminal proteome along a competition gradient in Drosophila melanogaster. Proc Natl Acad Sci U S A.

[22] Tuni C, Weber S, Bilde T, Uhl G (2017). Male spiders reduce pre- and postmating sexual investment in response to sperm competition risk. Behav Ecol.

[23] Bretman A, Fricke C, Hetherington P, Stone R, Chapman T (2010). Exposure to rivals and plastic responses to sperm competition in Drosophila melanogaster. Behav Ecol.

[24] Wang H, Duclot F, Liu Y, Wang Z, Kabbaj M (2013). Histone deacetylase inhibitors facilitate partner preference formation in female prairie voles. Nat Neurosci.

[25] Bird A (2007). Perceptions of epigenetics. Nature.

[26] Todd EV, Black MA, Gemmell NJ (2016). The power and promise of RNA-seq in ecology and evolution. Mol Ecol.

[27] Bloch NI (2018). Early neurogenomic response associated with variation in guppy female mate preference. Nat Ecol Evol.

[28] Bentz AB (2021). Experimental competition induces immediate and lasting effects on the neurogenome in free-living female birds. Proc Natl Acad Sci U S A.

[29] Anderson AP, Rose E, Flanagan SP, Jones AG (2020). The Estrogen-Responsive Transcriptome of Female Secondary Sexual Traits in the Gulf Pipefish. J Hered.

[30] Schunter C, Vollmer SV, Macpherson E, Pascual M (2014). Transcriptome analyses and differential gene expression in a non-model fish species with alternative mating tactics. BMC Genomics.

[31] Bukhari SA (2017). Temporal dynamics of neurogenomic plasticity in response to social interactions in male threespined sticklebacks. PLoS Genet.

[32] Eswine SL, Pontinen JK, Heimovics SA (2019). Competitive ability during mate competition relates to unique patterns of dopamine-related gene expression in the social decision-making network of male zebra finches. Neurosci Lett.

[33] Connahs H (2022). The yellow gene regulates behavioural plasticity by repressing male courtship in Bicyclus anynana butterflies. Proc Biol Sci.

[34] Aubin-Horth N, Landry CR, Letcher BH, Hofmann HA (2005). Alternative life histories shape brain gene expression profiles in males of the same population. Proc Biol Sci.

[35] Ramm SA (2019). Sex allocation plasticity on a transcriptome scale: Socially sensitive gene expression in a simultaneous hermaphrodite. Mol Ecol..

[36] Lilie ND, Riyahi S, Korsten P, Schmoll T. Male social niche conformance in zebra finches. 2019. OSF Preregistration. 10.17605/OSF.IO/84Z5R.

[37] Forstmeier W, Martin K, Bolund E, Schielzeth H, Kempenaers B (2011). Female extrapair mating behavior can evolve via indirect selection on males. Proc Natl Acad Sci U S A.

[38] Forstmeier W, Birkhead TR (2004). Repeatability of mate choice in the zebra finch: consistency within and between females. Anim Behav.

[39] Birkhead TR, Fletcher F, Pellatt EJ, Staples A (1995). Ejaculate quality and the success of extra-pair copulations in the zebra finch. Nature.

[40] Birkhead TR, Hunter FM, Pellatt JE (1989). Sperm competition in the zebra finch. Taeniopygia guttata Animal Behaviour.

[41] Lilie ND (2022). Male social niche conformance? Effects of manipulated opportunity for extra-pair mating on behavior and hormones of male zebra finches. Horm Behav.

[42] Zann RA (1996). The Zebra Finch: A Synthesis of Field and Laboratory Studies.

[43] Ruploh T, Bischof H-J, von Engelhardt N (2013). Adolescent social environment shapes sexual and aggressive behaviour of adult male zebra finches (Taeniopygia guttata). Behav Ecol Sociobiol.

[44] Ruploh T, Bischof H-J, von Engelhardt N (2014). Social experience during adolescence influences how male zebra finches (Taeniopygia guttata) group with conspecifics. Behav Ecol Sociobiol.

[45] Zimmermann TD, Kaiser S, Hennessy MB, Sachser N (2017). Adaptive shaping of the behavioural and neuroendocrine phenotype during adolescence. Proceedings of the Royal Society B: Biological Sciences.

[46] Arnold C, Taborsky B (2010). Social experience in early ontogeny has lasting effects on social skills in cooperatively breeding cichlids. Anim Behav.

[47] Hurley LL (2023). Longitudinal covariation of testosterone and sperm quality across reproductive stages in the zebra finch. Horm Behav.

[48] Goodson J (1997). Neurobiology of avian social organization. Effects of lateral septum lesions in a territorial songbird, the field sparrow (Spizella pusilla), and a colonial songbird, the zebra finch (Taeniopygia guttata). Ann N Y Acad Sci.

[49] Goodson JL (1998). Vasotocin and vasoactive intestinal polypeptide modulate aggression in a territorial songbird, the violet-eared waxbill (Estrildidae: Uraeginthus granatina). Gen Comp Endocrinol.

[50] Goodson JL, Eibach R, Sakata J, Adkins-Regan E (1999). Effect of septal lesions on male song and aggression in the colonial zebra finch (Taeniopygia guttata) and the territorial field sparrow (Spizella pusilla). Behav Brain Res.

[51] Herold C, Paulitschek C, Palomero-Gallagher N, Güntürkün O, Zilles K (2018). Transmitter receptors reveal segregation of the arcopallium/amygdala complex in pigeons (Columba livia). J Comp Neurol.

[52] Abellán A, Legaz I, Vernier B, Rétaux S, Medina L (2009). Olfactory and amygdalar structures of the chicken ventral pallium based on the combinatorial expression patterns of LIM and other developmental regulatory genes. J Comp Neurol.

[53] O’Connell LA, Hofmann HA (2011). The vertebrate mesolimbic reward system and social behavior network: a comparative synthesis. J Comp Neurol.

[54] Langfelder P, Horvath S (2008). WGCNA: an R package for weighted correlation network analysis. BMC Bioinformatics.

[55] Anders S, Pyl PT, Huber W (2015). HTSeq–a Python framework to work with high-throughput sequencing data. Bioinformatics.

[56] Kim D, Langmead B, Salzberg SL (2015). HISAT: a fast spliced aligner with low memory requirements. Nat Methods.

[57] Iancu OD, Colville A, Darakjian P, Hitzemann R (2014). Coexpression and cosplicing network approaches for the study of mammalian brain transcriptomes. Int Rev Neurobiol.

[58] Zhang B, Horvath S. A general framework for weighted gene co-expression network analysis. Stat Appl Genet Mol Biol. 2005;4:17. 10.2202/1544-6115.1128 16646834

[59] Ashburner M (2000). Gene ontology: tool for the unification of biology. The Gene Ontology Consortium Nat Genet.

[60] Tomczak A (2018). Interpretation of biological experiments changes with evolution of the Gene Ontology and its annotations. Sci Rep.

[61] Schmoll T, Kleven O, Rusche M (2018). Individual phenotypic plasticity explains seasonal variation in sperm morphology in a passerine bird. Evol Ecol Res.

[62] Frankl-Vilches C, Gahr M (2018). Androgen and estrogen sensitivity of bird song: a comparative view on gene regulatory levels. J Comp Physiol A Neuroethol Sens Neural Behav Physiol.

[63] Ball GF, Balthazart J (2010). Seasonal and hormonal modulation of neurotransmitter systems in the song control circuit. J Chem Neuroanat.

[64] Birkhead TR. Behavioral Aspects of Sperm Competition in Birds. Adv. Study Behav. 1988;18:35–72.

[65] Birkhead TR, Burke T, Zann R, Hunter FM, Krupa AP (1990). Extra-pair paternity and intraspecific brood parasitism in wild zebra finches Taeniopygia guttata, revealed by DNA fingerprinting. Behav Ecol Sociobiol.

[66] Griffith SC, Holleley CE, Mariette MM, Pryke SR, Svedin N (2010). Low level of extrapair parentage in wild zebra finches. Anim Behav.

[67] Birkhead TR, Pellatt EJ, Brekke P, Yeates R, Castillo-Juarez H (2005). Genetic effects on sperm design in the zebra finch. Nature.

[68] Mccarthy E, Mcdiarmid CS, Hurley LL, Rowe M, Griffith SC (2021). Highly variable sperm morphology in the masked finch (Poephila personata) and other estrildid finches. Biol J Lin Soc.

[69] Prior NH (2016). Sex steroid profiles and pair-maintenance behavior of captive wild-caught zebra finches (Taeniopygia guttata). J Comp Physiol A Neuroethol Sens Neural Behav Physiol.

[70] Prior NH (2017). Sex steroid profiles in zebra finches: Effects of reproductive state and domestication. Gen Comp Endocrinol.

[71] Perfito N, Zann RA, Bentley GE, Hau M (2007). Opportunism at work: habitat predictability affects reproductive readiness in free-living zebra finches. Funct Ecol.

[72] Mariette MM, Griffith SC (2015). The adaptive significance of provisioning and foraging coordination between breeding partners. Am Nat.

[73] Waas JR, Colgan PW, Boag PT (2005). Playback of colony sound alters the breeding schedule and clutch size in zebra finch (Taeniopygia guttata) colonies. Proc Biol Sci.

[74] Bogorad MI, DeStefano JG, Linville RM, Wong AD, Searson PC (2019). Cerebrovascular plasticity: Processes that lead to changes in the architecture of brain microvessels. J Cereb Blood Flow Metab.

[75] Phillips RE, Youngren OM (1986). Unilateral kainic acid lesions reveal dominance of right archistriatum in avian fear behavior. Brain Res.

[76] Morandi-Raikova A, Mayer U (2020). The effect of monocular occlusion on hippocampal c-Fos expression in domestic chicks (Gallus gallus). Sci Rep.

[77] Perez EC, Murisse M, Herve L, Georgelin M, Constantin P et al. Object and food novelty induce distinct patterns of c-fos immunoreactivity in amygdala and striatum in domestic male chicks (*Gallus gallus domesticus*). Behav Brain Res. 2020;381:112453. 10.1016/j.bbr.2019.112453 31883949

[78] Mayer U, Rosa-Salva O, Vallortigara G. First exposure to an alive conspecific activates septal and amygdaloid nuclei in visually-naïve domestic chicks (*Gallus gallus*). Behav Brain Res. 2016. 10.1016/j.bbr.2016.09.031 27633562

[79] Goodson JL (2005). The vertebrate social behavior network: evolutionary themes and variations. Horm Behav.

[80] Newman SW (1999). The medial extended amygdala in male reproductive behavior. A node in the mammalian social behavior network. Ann. N. Y. Acad. Sci.

[81] Colquitt BM, Merullo DP, Konopka G, Roberts TF, Brainard MS (2021). Cellular transcriptomics reveals evolutionary identities of songbird vocal circuits. Science..

[82] Hahnloser RHR, Kozhevnikov AA, Fee MS (2002). An ultra-sparse code underliesthe generation of neural sequences in a songbird. Nature.

[83] Leonardo A, Fee MS (2005). Ensemble Coding of Vocal Control in Birdsong. J Neurosci.

[84] Yu AC, Margoliash D (1996). Temporal hierarchical control of singing in birds. Science.

[85] Mello CV, Kaser T, Buckner AA, Wirthlin M, Lovell PV (2019). Molecular architecture of the zebra finch arcopallium. J Comp Neurol.

[86] Hahn LA, Balakhonov D, Fongaro E, Nieder A, Rose J. Working memory capacity of crows and monkeys arises from similar neuronal computations. eLife. 2021;10:e72783. 10.7554/eLife.72783 PMC8660017 34859781

[87] Güntürkün O, von Eugen K, Packheiser J, Pusch R (2021). Avian pallial circuits and cognition: A comparison to mammals. Curr Opin Neurobiol.

[88] Pusch R, Clark W, Rose J, Güntürkün O (2023). Visual categories and concepts in the avian brain. Anim Cogn.

[89] Lieshoff C, Große-Ophoff J, Bischof H-J (2004). Sexual imprinting leads to lateralized and non-lateralized expression of the immediate early gene zenk in the zebra finch brain. Behav Brain Res.

[90] Wylie DRW, Gutierrez-Ibanez C, Pakan JMP, Iwaniuk AN (2009). The optic tectum of birds: mapping our way to understanding visual processing. Can J Exp Psychol.

[91] developmental models of emerging animacy-detection mechanisms (2015). Rosa Salva, O., Mayer, U. & Vallortigara, G. Roots of a social brain. Neurosci Biobehav Rev.

[92] Clark WJ, Colombo M (2020). The functional architecture, receptive field characteristics, and representation of objects in the visual network of the pigeon brain. Prog Neurobiol.

[93] Bischof HJ, Watanabe S (1997). On the structure and function of the tectofugal visual pathway in laterally eyed birds. Eur J Morphol.

[94] Watanabe S, Mayer U, Bischof H-J (2011). Visual Wulst analyses ‘where’ and entopallium analyses ‘what’ in the zebra finch visual system. Behav Brain Res.

[95] Knudsen EI (2020). Evolution of neural processing for visual perception in vertebrates. J Comp Neurol.

[96] Woolley SMN, Casseday JH (2004). Response properties of single neurons in the zebra finch auditory midbrain: response patterns, frequency coding, intensity coding, and spike latencies. J Neurophysiol.

[97] Birkhead TR, Fletcher F, Pellatt EJ (1998). Sexual selection in the zebra finch Taeniopygia guttata : condition, sex traits and immune capacity. Behav Ecol Sociobiol.

[98] Nixdorf-Bergweiler BE, Bischof HJ. A Stereotaxic Atlas Of The Brain Of The Zebra Finch, *Taeniopygia Guttata*: With Special Emphasis on Telencephalic Visual and Song System Nuclei in Transverse and Sagittal Sections. Bethesda: National Center for Biotechnology Information. 2007.

[99] Chen Y (2018). SOAPnuke: a MapReduce acceleration-supported software for integrated quality control and preprocessing of high-throughput sequencing data. Gigascience.

[100] Andrews, S. *et al.* FastQC a quality control tool for high throughput sequence data. https://www.bioinformatics.babraham.ac.uk/projects/fastqc/. 2010.

[101] Love MI, Huber W, Anders S (2014). Moderated estimation of fold change and dispersion for RNA-seq data with DESeq2. Genome Biol.

[102] R Core Team (2020). R: A language and environment for statistical computing. R Foundation for Statistical Computing, Vienna, Austria. 2020. https://www.R-project.org/.

[103] Kanehisa M, Goto S (2000). KEGG: kyoto encyclopedia of genes and genomes. Nucleic Acids Res.

[104] Raudvere U (2019). g:Profiler: a web server for functional enrichment analysis and conversions of gene lists (2019 update). Nucleic Acids Res.

[105] Supek F, Bošnjak M, Škunca N, Šmuc T (2011). REVIGO summarizes and visualizes long lists of gene ontology terms. PLoS ONE.

